# Comparison of two drug combinations in total intravenous anesthesia: Propofol–ketamine and propofol–fentanyl

**DOI:** 10.4103/1658-354X.65132

**Published:** 2010

**Authors:** Sukhminder Jit Singh Bajwa, Sukhwinder Kaur Bajwa, Jasbir Kaur

**Affiliations:** *Department of Anaesthesiology & Intensive Care, Gian Sagar Medical College & Hospital, Ram Nagar, Banur, Punjab, India*; 1 *Department of Obstetrics & Gynaecology, Gian Sagar Medical College & Hospital, Ram Nagar, Banur, Punjab, India*

**Keywords:** *Propofol-ketamine*, *propofol-fentanyl*, *total intravenous anesthesia*

## Abstract

**Background and Aims:**

Keeping in consideration the merits of total intravenous anesthesia (TIVA), a genuine attempt was made to find the ideal drug combinations which can be used in general anesthesia. This study was conducted to evaluate and compare two drug combinations of TIVA using propofol–ketamine and propofol–fentanyl and to study the induction, maintenance and recovery characteristics following anesthesia with these techniques.

**Settings and Design:**

A case control study was conducted, which included 100 patients, in the department of Anaesthesiology and Intensive care, Government Medical College and Hospital, Patiala.

**Patients and Methods:**

A hundred patients between the ages of 20 and 50 years of either gender were divided into two groups of 50 each, and they underwent elective surgery of approximately 1 h duration. Group I received propofol–ketamine while group II received propofol–fentanyl for induction and maintenance of anesthesia. All the results were tabulated and analyzed statistically with student's unpaired *t*-test and chi-square test.

**Results:**

Propofol–fentanyl combination produced a significantly greater fall in pulse rate (PR; 9.28% versus 0.23%) and in both systolic (7.94% versus 0.12%) and diastolic blood pressures (BP; 8.10% versus 0.35%) as compared to propofol–ketamine during induction of anesthesia. Propofol–ketamine combination produced stable hemodynamics during maintenance phase while on the other hand propofol–fentanyl was associated with a slight increase in both PR and BP. During recovery, ventilation score was better in group I while movement and wakefulness score was better in group II. Mean time to protrusion of tongue and lifting of head was shorter in group I.

**Conclusions:**

Both propofol–ketamine and propofol–fentanyl combinations produce rapid, pleasant and safe anesthesia with only a few untoward side effects and only minor hemodynamic effects.

## INTRODUCTION

General anesthesia should provide quick and pleasant induction, predictable loss of consciousness, stable operating conditions, minimal adverse effects, rapid and smooth recovery of protective reflexes and psychomotor functions.

This study was conducted to evaluate and compare two drug combinations of TIVA using propofol–ketamine and propofol-fentanyl and to study the induction characteristics, maintenance of anesthesia and recovery characteristics following anesthesia with these techniques.

The development of anesthesia since its introduction has been erratic, long periods of stagnation being occasionally broken by improvement and advances. General anesthesia has undergone a vast number of improvements and modifications and even its recently modified form total intravenous anesthesia (TIVA; induction as well as maintenance of anesthesia with intravenous agents only) has undergone many improvements ever since its introduction into clinical practice.

Till recently, inhalational agents have remained the routine choice for maintenance of anesthesia. One of the principle reasons is the availability of sophisticated delivery systems for volatile anesthetics, which allows the anesthetists to have a fine degree of control on the concentration administered to the patient. Moreover, monitoring systems that permit nearly accurate measurement of end-tidal concentration of the volatile anesthetics as well as the introduction of new potent volatile agents provide a wider choice of drugs.

In spite of all these advantages, inhalational agents have their own drawbacks and shortcomings that are as follows:

- cost factor
- different specific vaporizers require repeated maintenance
- scavenging system is necessary; otherwise pollution of operation room environment is a big hazard.

TIVA has many advantages over inhalational anesthesia such as

- no operating room pollutions
- minimal cardiac depression
- lesser neurohumoral response
- decreased oxygen consumption
- avoids distension of air-filled spaces within the patient's body, thus producing optimum operating conditions for the surgeon
- avoids postoperative diffusion hypoxemia
- decreases the incidence of postoperative nausea and vomiting (PONV)
- In day care surgery, etc.

Moreover, TIVA can be used not only in well-equipped hospital setting but at remote location also with only oxygen and ventilation facilities.

Various drugs have been tried from time to time in TIVA. Since no single drug can provide all the characteristics of an ideal intravenous agent, several drugs are used in different combinations to provide balanced anesthesia in TIVA, that is, amnesia, hypnosis and analgesia.

In the lookout for an ideal intravenous anesthetic agent in clinical practice, Kay and Rolly introduced propofol in 1977.[1] Its advantage in short surgical procedures relates to its rapid elimination from the blood (half life 1–3 h due to high hepatic clearance) leading to rapid recovery of cognitive and psychomotor functions with a very low incidence of PONV. It is primarily a hypnotic and in subhypnotic doses provides sedation and amnesia. Lack of analgesic properties of propofol has necessitated the need for supplementary analgesic agents during TIVA. Morphine and pethidine have been replaced by newer agents such as fentanyl, sufentanyl, alfentanyl and remifentanyl, which can be given either in multiple bolus incremental doses or in continuous infusion form. Ketamine in subanesthetic doses has gained more attention as an analgesic for TIVA.[2]

Fentanyl is used extensively in TIVA now-a-days. It belongs to opioid group of drugs. It is hundred times more potent analgesic than morphine, and as a part of balanced anesthesia it relieves pain, reduces somatic and autonomic response to airway manipulation, provides hemodynamic stability and lesser respiratory depression.[3]

The combination of these drugs provides complete and balanced anesthesia and has advantages such as high potency, lower dosages and fewer side effects. In the quest for complete anesthesia, various combinations of these new drugs have been tried which include midazolam–ketamine, propofol–ketamine, propofol–fentanyl and many more each with varying results.

Keeping in consideration the merits of TIVA, a case control study was conducted on 100 patients in Department of Anaesthesiology and Intensive care, Government Medical College and Hospital, Patiala.

## PATIENTS AND METHODS

One hundred adult patients of age between 20 and 60 years and of ASA grade I or II of either sex who underwent elective surgery at Government Medical College and Hospital, Patiala, were included in the study. Patients were divided randomly into two groups of 50 patients each depending upon the drug combinations used.

Following patients were excluded from study: history of allergy to any particular drug, allergy to egg or fat, pregnant females, patients on Monoamine oxidase inhibitors, history of jaundice, extremes of age, duration of surgery lasting for more than 80 minutes.

As a premedication, tablets ranitidine 150 mg + alprazolam 0.25 mg were given, a night before and 2 h before the induction of anesthesia.

### Anesthetic technique

Standard anesthetic technique was used in all the patients. After securing intravenous line, monitoring gadgets were attached which included ECG, SpO_2_ and noninvasive BP cuff. Baseline parameters were observed and recorded. Injection midazolam (0.08 mg/kg with maximum dose of 5 mg) was given IV 2 minutes before the induction of anesthesia in both the groups.

### Induction of anesthesia

Induction of anesthesia in patients of group I was done with propofol 1.0 mg/kg body wt. and ketamine 1.0 mg/kg body wt. given as IV bolus doses. In group II, induction of anesthesia was done with propofol 1.5 mg/kg body wt. and fentanyl 2.0 µg/kg body wt. given as IV bolus doses.

In both the groups, injection succinylcholine was given as a muscle relaxant before intubation in doses of 1.5 mg/kg body wt. with maximum doses not exceeding 100 mg. Patients were ventilated with 100% oxygen via a facemask for 60–90 seconds with the help of Bains circuit, and intubation was done with an appropriate size of cuffed endotracheal tube. Hemodynamic and other monitoring parameters were observed continuously and recorded at an interval of 1 minute each for the first 5 minutes.

### Maintenance of anesthesia

In group I, maintenance of anesthesia was achieved with infusion of propofol 2.0 mg/kg/h and ketamine 2.0 mg/kg/h, while in group II, maintenance of anesthesia was achieved with infusion of propofol 2.0 mg/kg/h and fentanyl 2.0 µg/kg/h.

Vecuronium bromide was used as a muscle relaxant in doses of 0.05–0.06 mg/kg body wt. as an initial bolus dose and supplemented with top-ups of 1 mg in both the groups. Hemodynamic and other monitoring parameters were observed continuously and noted at an interval of 5 minutes during the operation. Patients were ventilated with 100% oxygen with close circuit attached to circle absorber system.

### Reversal of relaxant effect

All the anesthetic drugs were stopped 5–7 minutes before the anticipated end of surgery. At the end of surgery, neuromuscular blockade was reversed with injection neostigmine 40 µg/kg body wt. and injection glycopyrrolate 10 µg/kg body wt. which was given over 2–3 minutes. Extubation was done when the patients were able to maintain rhythmic respiration and adequate tidal volume. The monitoring parameters were observed continuously and recorded at the time of extubation and 5 minutes after that. The parameters were again recorded every 15 minutes in the recovery room.

## RESULTS

All the results were tabulated and analyzed statistically with student's unpaired *t*-test and chi-square test.

Four patients (8%) from group I and five patients (10%) from group II had involuntary movements during the induction of anesthesia.

### Pulse rate

There was an increase in PR in group I, while there was a slight decrease in PR in group II patients after induction of anesthesia which returned gradually toward baseline during the maintenance phase of anesthesia in both the groups, but the difference in both the groups was statistically significant (*P* < 0.05). PR increased in both the groups at 1 and 5 minutes after extubation [Table 1].

**Table 1:** Comparison of mean pulse rate of both the groups at different stages of anesthesia in group I and group II

Anesthesia stage	Time interval	Group	Mean ± SD	*t*	*P*	Significance
Preinduction		I	84.08 ± 5.18	‐	>0.05	NS
		II	84.18 ± 5.28			
Induction	1 M	I	84.28 ± 5.20	20.90	<0.05	S
		II	76.36 ± 4.52			
Intubation	2 M	I	90.64±5.30	24.97	<0.05	S
		II	78.36 ± 4.40			
	3 M	I	90.84 ± 5.14	26.52	<0.05	S
		II	77.32 ± 4.36			
	4 M	I	90.72 ± 5.20	26.17	<0.05	S
		II	77.20 ± 4.29			
	50 M	I	90.76 ± 5.31	27.12	<0.05	S
		II	76.96 ± 4.33			
Intraoperative	10 M	I	86.32 ± 5.11	3.56	<0.05	S
		II	85.20 ± 4.31			
	20 M	I	84.52 ± 5.32	15.31	<0.05	S
		II	88.0 ± 4.79			
	30 M	I	84.32 ± 5.08	15.47	<0.05	S
		II	87.68 ± 4.64			
	40 M	I	84.92 ± 5.19	7.31	<0.05	S
		II	87.32 ± 4.52			
	50 M	I	84.56 ± 5.09	7.69	<0.05	S
		II	87.07 ± 4.48			
	60 M	I	84.28 ± 5.04	9.35	<0.05	S
		II	87.36 ± 4.12			
Postoperative	1 M	I	86.56 ± 4.96	7.97	<0.05	S
		II	89.24 ± 3.97			
	5 M	I	84.36 ± 5.12	2.30	<0.05	S
		II	85.32 ± 4.05			
	10 M	I	84.28 ± 5.29	1.19	>0.05	NS
		II	84.80 ± 4.00			
	15 M	I	84.40 ± 5.39	0.68	>0.05	NS
		II	84.04 ± 4.85			
	20 M	I	84.56 ± 5.43	0.90	>0.05	NS
		II	84.12 ± 5.11			

### Blood pressure

There was a fall in BP (systolic and diastolic) during the induction of anesthesia in group II, while there was a slight increase in BP in group I after induction and intubation which was statistically significant (*P* < 0.05). During maintenance there was gradual recovery toward baseline. During recovery period in both the groups, the BP increased again (1 minute after extubation), which was statistically significant (*P* < 0.05) but returned toward baseline in the next 20 minutes [Tables 2 and 3].

**Table 2:** Comparison of systolic blood pressure of both the groups at different stages of anesthesia in group I and group II

Anesthesia stage	Time interval	Group	Mean ± SD	*t*	*P*	Significance
Preinduction		I	125.96 ± 9.55	0.22	>0.05	NS
		II	126.40 ± 9.67			
Induction	1M	I	125.80 ± 9.26	5.01	<0.05	S
		II	116.36 ± 9.50			
Intubation	2M	I	136.08 ± 9.67	7.31	<0.05	S
		II	122.16 ± 9.31			
	3 M	I	135.68 ± 9.64	7.57	<0.05	S
		II	121.28 ± 9.37			
	4 M	I	132.04 ± 9.68	5.73	<0.05	S
		II	121.08 ± 9.42			
	5M	I	130.28 ± 9.47	5.37	<0.05	S
		II	120.20 ± 9.27			
Intraoperative	10M	I	129.76 ± 9.47	4.83	<0.05	S
		I	126.20 ± 9.20			
	20 M	I	128.66 ± 9.84	3.98	<0.05	S
		II	130.28 ± 9.14			
	30 M	I	128.24 ± 9.73	5.40	<0.05	S
		II	132.04 ± 8.59			
	40 M	I	128.04 ± 9.82	3.03	<0.05	S
		II	130.24 ± 8.47			
	50 M	I	127.92 ± 0.36	5.39	<0.05	S
		II	132.08 ± 8.53			
Intraoperative	60 M	I	127.88 ± 9.27	7.97	<0.05	S
		II	134.10 ± 8.52			
Postoperative	1 M	I	132.24 ± 9.65	5.57	<0.05	S
		II	136.12 ± 8.52		
	5 M	I	128.36 ± 9.79	0.06	>0.05	NS
		II	128.32 ± 9.25			
	10 M	I	128.24 ± 9.74	0.29	>0.05	NS
		II	126.28 ± 9.18			
	15 M	I	128.04 ± 9.60	0.63	>0.05	NS
		II	125.20 ± 9.23			
	20 M	I	127.80 ± 9.60	0.94	>0.05	NS
		II	125.64 ± 6.28			

**Table 3:** Comparison of diastolic blood pressure of both the groups at different stages of anesthesia in group I and group II

Anesthesia stage	Time interval	Group	Mean ± SD	*t*	*P*	Significance
Preinduction		I	80.54 ± 3.56	0	>0.05	NS
		II	80.09 ± 3.55			
Induction	1M	I	80.32 ± 3.55	9.62	<0.05	S
		II	73.60 ± 3.61			
Intubation	2M	I	86.24 ± 3.76	16.30	<0.05	S
		II	75.72 ± 3.54			
	3 M	I	86.68 ± 3.86	11.78	<0.05	S
		II	75.48 ± 3.44			
	4 M	I	86.44 ± 3.73	12.30	<0.05	S
		II	75.32 ± 3.53			
	5M	I	86.92 ± 3.55	11.01	<0.05	S
		II	75.20 ± 3.47			
Intraoperative	10M	I	81.84 ± 3.63	2.83	<0.05	S
		I	81.12 ± 3.57			
	20 M	I	81.32 ± 3.99	7.43	<0.05	S
		II	83.48 ± 3.51			
	30 M	I	81.28 ± 3.95	10.25	<0.05	S
		II	84.44 ± 3.52			
	40 M	I	81.44 ± 4.04	6.90	<0.05	S
		II	83.96 ± 3.39			
	50 M	I	81.36 ± 4.32	7.87	<0.05	S
		II	84.84 ± 3.38			
Intraoperative	60 M	I	81.52 ± 3.90	10.04	<0.05	S
		II	85.24 ± 3.35			
Postoperative	1 M	I	82.04 ± 4.03	11.10	<0.05	S
		II	86.36 ± 4.17		
	5 M	I	79.12 ± 3.85	2.27	>0.05	NS
		II	80.84 ± 3.57			
	10 M	I	78.72 ± 4.11	2.95	>0.05	NS
		II	80.40 ± 3.42			
	15 M	I	78.60 ± 4.33	2.58	>0.05	NS
		II	79.80 ± 3.00			
	20 M	I	78.56 ± 4.21	2.58	>0.05	NS
		II	79.76 ± 3.53			

### SPO_2_

It was found in both the groups that there was very little change in mean SPO_2_ values during induction and maintenance of anesthesia as well as during recovery phase.

### Recovery

Ventilation score was better in group I during the first 10 minutes of recovery phase as compared to group II [Table 4].

- Mean movement score was better in group II at 5 and 10 minutes [Table 5].
- Wakefulness score was better in group II at 5 and 10 minutes as compared to group I [Table 6].
- The mean time for appearance of protective airway reflexes (coughing and gagging), spontaneous eye opening, tongue protrusion and lifting of head was shorter in group II [Table 7].
- One patient (2%) from group I and three patients (6%) from group II had nausea during the recovery phase while none of them had any episode of vomiting.

Secretions: In group II, four patients had oral secretions during recovery from anesthesia.

**Table 4:** Recovery (ventilation score) of both the groups

Time interval	Group	Mean ± SD	Difference	*t*	*P*	Significance
1 M	I	0.06 ± 0.23	0.06	1.76	>0.05	NS
	II	0.0			
5 M	I	0.84 ± 0.37	0.26	2.96	<0.05	S
	II	0.58 ± 0.49				
10 M	I	1.90 ± 0.30	0.40	4.32	<0.05	S
	II	1.50 ± 0.58			
15 M	I	1.94 ± 0.23	0.08	1.33	>0.05	NS
	II	1.86 ± 0.55			
20 M	I	2.0 ± 0	0.06	1.76	>0.05	NS
	II	1.94 ± 0.25			

**Table 5:** Recovery (movement score) of both the groups

Time interval	Group	Mean ± SD	Difference	*t*	*P*	Significance
1 M	I	0.0 ± 0	0.06	1.76	>0.05	NS
	II	0.06			
5 M	I	0.48 ± 0.50	0.30	3.23	<0.05	S
	II	0.78 ± 0.41				
10 M	I	1.20 ± 0.49	0.30	3.0	<0.05	S
	II	1.50 ± 0.50			
15 M	I	1.90 ± 0.30	0	0	>0.05	NS
	II	1.96 ± 0.19			
20 M	I	2.0 ± 0	0.06	1.76	>0.05	NS
	II	2.0 ± 0			

**Table 6:** Recovery (wakefulness score) of both the groups

Time interval (minutes)	Group	Mean ± SD	Difference	*t*	*P*	Significance
1 M	I	0.0 ± 0	0	0	>0.05	NS
	II	0 ± 0			
5 M	I	0.44 ± 0.50	0.20	2.02	<0.05	S
	II	0.64 ± 0.48				
10 M	I	0.80 ± 0.49	0.24	2.97	<0.05	S
	II	1.04 ± 0.28			
15 M	I	1.68 ± 0.47	0.02	0.21	>0.05	NS
	II	1.70 ± 0.46			
20 M	I	1.94 ± 0.23	0.06	1.76	>0.05	NS
	II	2.0 ± 0			

**Table 7:** Postoperative side effects in both the groups

Side effects	Group I		Group II	
	No.	% Age	No.	% Age
Nausea	1	2	3	6
Vomiting	—	—	—	—
Secretions	4	8.00	—	—
Laryngospasm/bronchospasm	—	—	—	—
Venous sequelae	—	—	—	—
Post-ketamine sequelae				
Excitation	2	4.00	—	—
Hallucinations	—	—	—	—
Euphoria	—	—	—	—
Any other	—	—	—	—

Post-ketamine sequelae: Two patients (4%) from group I had excitation postoperatively while none of the patients from group II had excitation or any other post-ketamine sequelae like dreams, hallucinations, euphoria, etc.

## DISCUSSION

The same dosages of propofol and fentanyl have greater impact on elderly as compared to young patients.[4] In older patients, the total dose of propofol administered decreases while other demographic features did not have any effect.[5]

The demographic profile of this study was almost similar to many studies except that of Nielsen *et al*, which showed greater impact on hemodynamic parameters in elderly patients as compared to young patients. This difference can be attributed to the selection of older age group in their study and they used a higher dose of fentanyl 4 µg/kg as compared to the use of 2.0 µg/kg of fentanyl in this study.

As far as the hemodynamic parameters are concerned, there was a slight decrease in heart rate (9%) in propofol–fentanyl group as compared to propofol–ketamine combination in the study of Mayer *et al*,[6] and Mi *et al*.[7] Studies of Mi *et al*, also showed that after induction, the PR did not alter significantly when propofol was used alone but decreased between 5 and 35% in patients who were given fentanyl 4 µg/kg prior to the induction of anesthesia.[4,7]

The results of this study are consistent with those obtained in the studies of Mayer and Mi. Increase in heart rate with propofol and ketamine can be explained on the basis of

- cardio stimulant effect of ketamine
- stress response during intubation.

The combination of propofol with fentanyl leads to decrease in heart rate due to the prevention of stress response by fentanyl and and its myocardial depressing effect.

Mi *et al*, observed greater hemodynamic and electroencephalograph responses to intubation in patients who received propofol than in those who received both propofol and fentanyl (*P* < 0.05). Hernandez *et al*,[8] carried out a study with propofol–ketamine, midazolam–ketamine and propofol–fentanyl combinations and observed stable hemodynamics in patients who received propofol and ketamine, whereas patients who had received midazolam–ketamine had significantly higher number of hypertensive peaks. In this study, the increase in mean systolic and diastolic BP in group I patients at 2 minutes may be due to the cardiac stimulant effect of ketamine and mild stress response to intubation, while during induction, maintenance and recovery, BP remained near preinduction values mainly due to the antagonistic properties of propofol (decrease in BP) and ketamine (increase in BP). In group II patients, both the mean systolic and diastolic BP decreased during induction because of the additive action of propofol and fentanyl.[9] Whereas at 2 minutes (just after laryngoscopy and intubation), stress response was prevented mainly by the action of fentanyl. During recovery period, the increase in both systolic and diastolic BP (1 minute after extubation) in both the groups was mainly due to the awakening response to extubation.

The extent and degree of various *induction characteristics* like loss of consciousness (onset of sleep),[10] loss of eyelash reflex[11] and apnoea during induction[10,12] showed quite a few similarities as well as differences from other studies and this may be probably due to the variations in the dosages as well as combinations of anesthetic drugs used.

The incidence of side effects like excitatory movements (hiccups, hypertonus, twitching or tremors) was higher with propofol alone during induction than when used in combination with fentanyl.[13] The differences from this study can be explained on the basis that they used propofol alone and that too in higher doses. Pain at injection site, cough and involuntary movements during induction of anesthesia,[5,14] were present to a lesser degree in this study, and the differences can be ascribed to diminishing of the excitatory effects of propofol at low doses and suppression of excitatory effects by fentanyl and ketamine. Similarly, absence of cough was due to lower dose (2 µg/kg) of fentanyl which was analgesic dose and not the induction dose.

### Recovery

A striking feature of the use of these drug combinations in TIVA has been the early recovery. In our study, two methods of recovery from anesthesia have been used.

The first method is the Steward Scoring System[15] which evaluates the recovery from anesthesia by physical evaluation (ventilation, movement, wakefulness). There was slight respiratory depression postoperatively in patients who received propofol–fentanyl as compared to patients who received propofol–ketamine. The slightly lower ventilation score with propofol–fentanyl combination was due to central respiratory depressant effect of fentanyl.[6,16] Movement score was better in group II as shown by the earlier recovery of voluntary movements in patients as compared to group I patients and were most probably due to longer sedative action of ketamine which leads to late return of voluntary movements.[6] Better wakefulness score in group II may be due to shorter duration of action of fentanyl as compared to ketamine which has increased sedating effect.[8]

The second method of evaluation of recovery which was used in this study was by observing the *return of protective airway reflexes* like coughing and gagging and *response to verbal commands* like spontaneous opening of eyes, protrusion of tongue and lifting of head. Spontaneous recovery was achieved much earlier in the propofol–fentanyl group as compared to the propofol–ketamine group. Except for slight respiratory depression which was caused by fentanyl, better recovery score in group II was most probably due to lesser sedative effects of fentanyl as compared to ketamine.[16–20]

### Side effects during recovery

The increased incidence of oral secretions in four patients of group I as compared to none in group II postoperatively may be due to the salivatory effect of ketamine. Slightly higher incidence of nausea in group II may be due to the central emetic effects of fentanyl.[21] But, as a whole, lower incidence of nausea and no incidence of vomiting are attributed to the antiemetic effect of propofol. This is all the more important at low doses and we have used propofol in low doses in this study. Propofol has been used successfully to treat postoperative nausea in a bolus dose of 10 mg and has been successfully used to treat refractory PONV.

Two patients (4%) from group I had excitation postoperatively while no patient from group II had this side effect, and this can be explained on the basis of lower dosage of ketamine used (1 mg/kg) in this study.[8] There were no other complication like awareness, mood changes, agitation, and all the patients were satisfied with the anesthetic technique used and described it as pleasant.

## CONCLUSIONS

In conclusion, the results of this study suggest that both propofol–ketamine and propofol–fentanyl combinations produce rapid, pleasant and safe anesthesia with only a few untoward side effects and only minor hemodynamic fluctuations. Although propofol–fentanyl combination produced hypotension during induction of anesthesia, it prevented stress–response during laryngoscopy and intubation. Propofol–ketamine combination produced stable hemodynamics during maintenance phase, while on the other hand propofol–fentanyl was associated with slight increase in both PR and BP during maintenance phase. There was a slight respiratory depression during recovery in patients who received propofol–fentanyl as was evident from the ventilation score. But on the other hand other recovery characteristics like awakening time and response to verbal commands were better in the propofol–fentanyl group. However, as far as recovery is concerned, one of the most important areas in evaluating day care surgical procedures, both propofol–ketamine and propofol–fentanyl are associated with smooth and swift recovery with minimal residual impairment of mental functioning which are due to their significant metabolism, short elimination half life and extremely high total body clearance.

So it may be recommended that both propofol–ketamine and propofol–fentanyl can be used as an excellent combination in TIVA for both elective and day care surgery where minimal side effects and early recovery are desired.
